# Defect generation in Pd layers by ‘smart’ films with high H-affinity

**DOI:** 10.1038/s41598-017-09900-z

**Published:** 2017-08-25

**Authors:** Vladimir Burlaka, Vladimir Roddatis, Marian David Bongers, Astrid Pundt

**Affiliations:** Universität Göttingen, Institut für Materialphysik, Friedrich-Hund-Platz 1, 37077 Göttingen, Germany

## Abstract

In this paper, we demonstrate that the microstructure and the surface of a thin palladium (Pd) film can be intentionally altered by the presence of a subjacent niobium (Nb) film. Depending on the thickness of the Nb film and on the hydrogen gas pressure, defects in the Pd film can be healed or created. To demonstrate this effect, Pd/Nb/sapphire (Al_2_O_3_) stacks are studied during hydrogen gas exposure at room temperature by using scanning tunneling microscopy (STM), X-ray diffraction (XRD) and environmental transmission electron microscopy (ETEM). STM shows that hydrogen-induced topography changes in the Nb films depend on the film thickness which affects the height of the Nb surface corrugations, their lateral size and distribution. XRD measurements show that these changes in the Nb hydride film influence the microstructure of the overlaying Pd film. ETEM reveals that the modifications of the Pd film occur due to the precipitation and growth of the Nb hydride phase. The appearance of new defects, interface and surface roughening is observed in the Pd film above locally grown Nb hydride grains. These results can open a new route to design ‘smart’ catalysts or membranes, which may accommodate their microstructure depending on the gaseous environment.

## Introduction

Nano-sized metal films are widely used as a key element for hydrogen storage^1, 2^, hydrogen gas sensors^3–5^, gas purification membranes^6, 7^ and heterogeneous catalysts^8, 9^. The surface morphology in such films plays an important role, especially for membrane applications and catalytic reactions, as edges and corners can work as active sites^10^. In the presence of gases the surface can reconstruct, creating added rows and valleys of atoms^11–14^. Moreover, as recently reported by Teschner *et al*. and Aleksandrov *et al*., the underlying material (support material) can also influence surface catalytic reactions^8, 15, 16^. In turn reaction products may dissolve in metallic films and particles as well, and thereby alter the electronic structure of the material and the surface reactivity^17–19^.

Among other metals palladium (Pd) is of particular interest because of its high catalytic activity^6, 7, 20, 21^. Dissolution of atomic hydrogen (H) in the Pd subsurface region and interior is observed even at low H_2_ partial pressures. While the solubility in surface and subsurface regions can reach up to 1 H/Pd^22–24^, the solubility of hydrogen on interior interstitial Pd lattice sites is small for low H_2_ partial pressures (in the Pd-H α-phase: less than 0.02 H/Pd). Thus, the mean hydrogen solubility depends on the Pd-particle size or the Pd film thickness and, additionally, on the microstructure^25–27^. The relatively low hydrogen content in Pd results in small hydrogen-induced mechanical stress and strain^27^. Compared to Pd, the solubility of H in niobium (Nb) is several orders of magnitude higher, reflecting the more negative enthalpy of hydrogen solution of −0.35 eV/atom in Nb^28^ compared to +0.1 eV/atom in Pd^29^. Nb-H forms a hydride already at about p_H2_ ≈1 × 10^−6^ mbar at 293 K^30^, whereas the formation pressure of Pd hydride is much higher - p_H2_ ≈ 18 mbar at 293 K^31^. Thus, in case of combined Pd/Nb - system, preferential hydrogen absorption at p_H2_ ≪18 mbar occurs first in Nb, at 293 K. If the hydrogen concentration is large, hydrogen absorption in thin metal films grown on rigid substrates can cause high mechanical stresses and strong out-of-plane lattice expansion^27, 32–36^. The growth of hydride precipitates within the Nb films gives rise to local lattice expansions and surface roughening.

Hydrogen absorption of 1 H/Nb in epitaxial Nb (110) films on rigid substrates results in a strong out-of-plane expansion^30, 33, 37, 38^ and a considerable mechanical stress up to 10 GPa^39^. If the initial thickness of the Nb film is *d*_*Nb*_ < 5 nm, stress release is not possible and an elastic stress evolution is measured up to a solubility of 1 H/Nb^39^. In films with a maximum initial thickness of *d*_*Nb*_ ≅ 9 ± 1 nm, the accumulated mechanical stress results in such a strong destabilization of the hydride phase that the phase transformation is suppressed even at 293 K^30^. Films with supressed phase transformation do not show any hydride-related topography changes.

However, above an initial Nb-film thickness of 10 nm, precipitation and growth of the hydride phase is observed and topography changes appear. The precipitation and growth mechanism depends on the film thickness^37, 40^, as the coherency between the hydride and the α-matrix changes. Precipitates are coherent with the matrix for 10 nm < *d*_0_ ≤ 37 nm^40^ and incoherent for *d*_0_ ≥ 40 nm^37^. For coherent precipitates, the lattice planes between the hydride precipitates and the α-matrix match by local strain, while for incoherent precipitates dislocations are present to adjust for the mismatch between the lattice planes^41^. The two states result in different surface topographies in the two-phase region of the Nb-H thin films system, with changes in lateral spreading as well as in the local topographical heights^33, 37^.

The landscape of the morphological changes on the Nb film is predicted by linear elastic theory^41, 42^., Therein, an out-of-plane film expansion *z* for a hydrogen-absorbing Nb-film grown on a rigid substrate is described by ${\rm{\Delta }}{z}_{theor}=0.136\cdot {\rm{\Delta }}{c}_{H}\cdot {d}_{o}$ ^29, 39^, where Δ*c*_*H*_ is the change of the hydrogen concentration within the film. This can be used to determine the surface topography changes expected for an incoherent phase transformation^41, 42^. In this case, Δ*c*_*H*_ corresponds to the width of the miscibility gap between two related phases. In the coherent regime, the out-of-plane expansion is less than expected in the incoherent regime (${\rm{\Delta }}{z}_{theor}$)^41^, thus it turns out that the amplitude of local topographic changes caused by hydrogen absorption can be maximized by choosing the incoherent regime (*d*_Nb_ > 40 nm). The amount and length (perimeter) of *bordering regions (modified regions of the interface)* can be tuned by changing the lateral spreading of hydrides. For example, a maximal length of bordering regions can be created by focusing on the coherent phase transformation regime.

In the present study, we demonstrate experimentally that the lattice expansion during hydrogen absorption in Nb-films modifies the microstructure of an overlying Pd layer in the Pd/Nb/ sapphire (Al_2_O_3_) system. Variation of the Nb-film (‘smart layer’) thickness allows for tuning of the out-of-plane expansion and the Pd/Nb interface roughness, causing local changes (*bending*) in the Pd top-layer. The border regions between the Nb α-phase and the Nb-hydride phase are suggested as origins for defect generation in the Pd top-layer and bending of its surface. We use a combination of *in situ* characterization techniques to support our hypothesis.

## Results and Discussion

**Figure 1:**
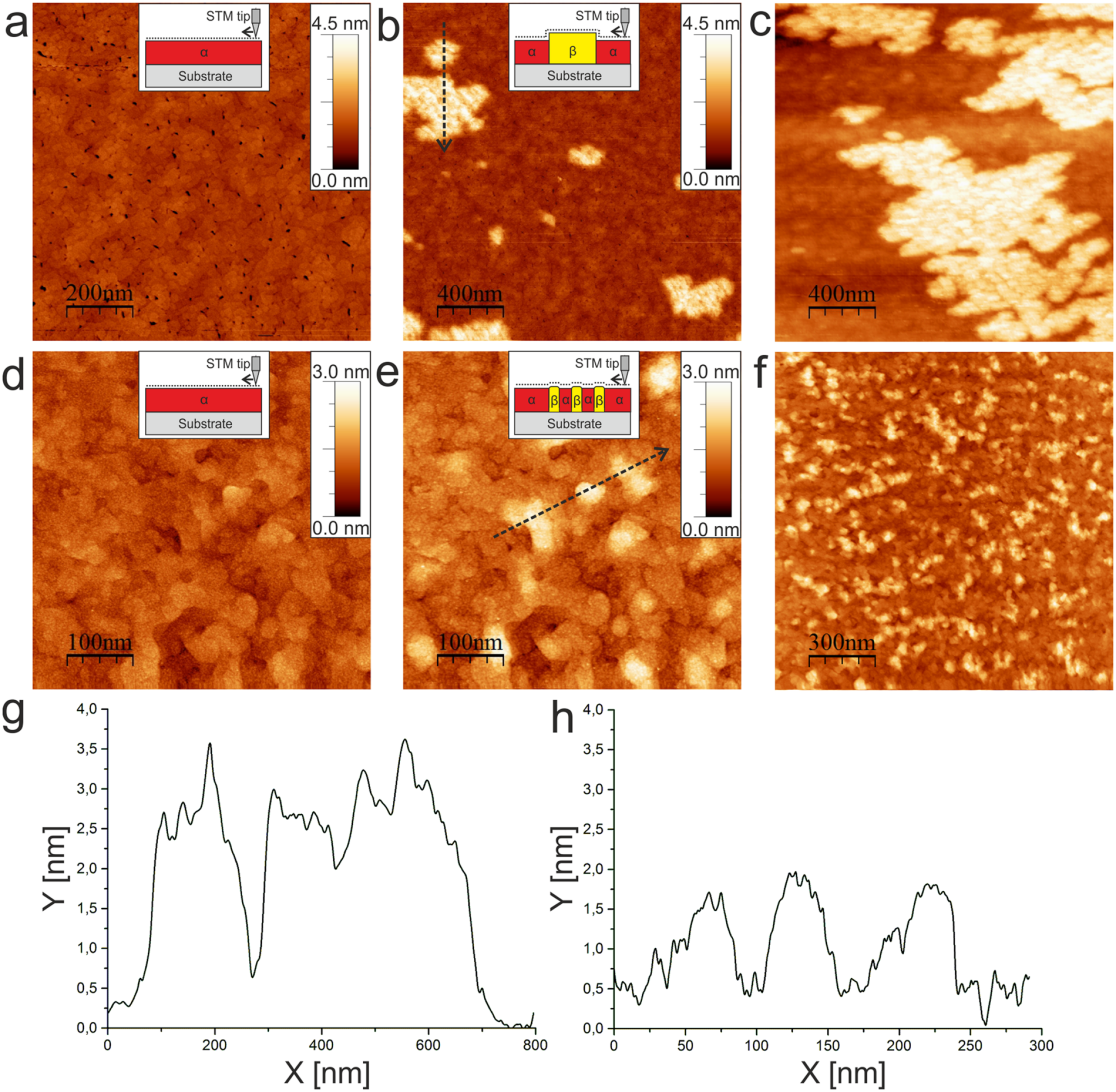
Hydrogen-induced surface topography changes of Pd/Nb/Al_2_O_3_ films measured *in situ* by STM. (**a–c**) Pd0.2nm/Nb40 nm/Al_2_O_3_: (**a**) before hydrogen exposure, (**b**) during hydrogen exposure at *p*_*H*_ = 1.6 × 10^−6^ mbar for 100 min, (**c**) during hydrogen exposure at *p*_*H2*_ = 3 × 10^−6^ mbar for 135 min. (**d**–**f**) Pd0.2nm/Nb25nm/Al_2_O_3_: (**d**) before hydrogen exposure; (**e**) during hydrogen exposure at *p*_*H2*_ = 7 × 10^−7^ mbar for 690 min; (**f**) during hydrogen exposure at *p*_*H2*_ = 1.6 × 10^−6^ mbar for 300 min. (**g**) Line scan taken along the black dotted lines in (**b**,**h**) line scan taken along the black dotted lines in (**e**) (Frame sizes a) 1000 × 1000 nm^2^, (**b**) 2000 × 2000 nm^2^, (**c**) 2000 × 2000 nm^2^, (**d**,**e**) 500 × 500 nm^2^, (**f**) 1500 × 1500 nm^2^).

The results of STM measurements performed during hydrogen gas exposure of a Pd0.2nm/Nb40nm/Al_2_O_3_ film (a)–(c), (g) in the incoherent regime, and a Pd0.2nm/Nb25nm/Al_2_O_3_ film (d)–(f), (h) in the coherent regime are shown in Fig. 1. Upon hydrogen gas exposure at *p*_*H*_ = 1.6 × 10^−6^ mbar, different hydride-related local topographical changes (bright regions) occur. Figure 1(a) shows the surface topography before the hydrogen exposure, Fig. 1(b) - during hydrogen exposure at *p*_*H*_ = 1.6 × 10^−6^ mbar (t_*e*_ = 100 min), Fig. 1(c) –during hydrogen exposure at *p*_*H*_ = 3 × 10^−6^ mbar (*t*_*e*_ = 135 min), and Fig. 1(g) – a line scan taken along the black dotted line shown in Fig. 1(b). The lateral size of the surface corrugations visible in Fig. 1(b) and (c) varies from 100 nm to about 1000 nm. We note that the lateral sizes and the distribution of initial film corrugations (dark dots in Fig. 1(a)) do not correlate with the lateral size and the distribution of the hydrides in Fig. 1(b). Thus, the initial film corrugation influence is here regarded as being of minor importance. The average distance between the neighboring elevated regions is large in Fig. 1(b), and ranges from 500 nm to about 750 nm. An example of a line scan taken across one of the hydrides shown in Fig. 1(b) is given in Fig. 1(g). The amplitude of surface corrugations in the hydride-related regions are on average about 2.2 nm. Linear elastic theory predicts 2.2 nm (with Δc_H_ = 0.4 H/Nb and d = 40 nm) for this film thickness, showing good agreement between the theory and the measurement.

For the 40 nm film, only a few relatively large hydride regions are detected in the area of interest. The chosen frame is representative of the complete film. Similar results were obtained for thicker Nb films^30, 33^. The perimeter of the border region between the hydride and the α-phase region can be determined by analyzing STM images measured at different loading stages. For example, the STM-image in Fig. 1(c) corresponds to a hydride volume fraction of about 41% (the volume fraction is estimated by the area fraction, as the hydrides in the films extend down to the substrate^41^). The relative length of the border region in Fig. 1(c) is 6.1∙10^−3^ nm^−1^.

Compared to the 40 nm film, STM results on the 25 nm film show a different precipitation and growth mode. The number of hydride regions drastically increases, while the typical lateral size decreases, as shown in Fig. 1(d–f). Here, Fig. 1(d) shows the surface topography before the hydrogen exposure, Fig. 1(e) shows the topography at *p*_*H*_ = 7 × 10^−7^ mbar (*t*_*e*_ = 690 min), Fig. 1(f) at *p*_*H*_ = 1.6 × 10^−6^ mbar (*t*_*e*_ = 300 min), and Fig. 1(h) provides the line scan taken along the black dotted line in Fig. 1(e). Hydride precipitates reach an average lateral size of about 50–100 nm, while the average distance between the hydrides varies in the range from 50–150 nm. The induced surface corrugations have an average height amplitude of about 1.3 nm (Fig. 1h). Linear elastic theory predicts a similar value of 1.4 nm for this film thickness (Δc_H_ = 0.4 H/Nb, d = 25 nm). This drastic change of morphology and distribution of hydrides compared to the thicker film is related to the different coherent state during the phase transformation^37, 40^.

Thus, the spatial distribution of hydride-induced surface corrugations as well as their typical height amplitude and lateral size can be changed by varying only the thickness of the Nb film. In comparison to the 40 nm film, the 25 nm film has a higher density of hydrides, as visible in Fig. 1(c and f). The volume fraction of the hydride phase in Fig. 1(f) is about 30% and the length of the border region is about 2.3 ∙ 10^−2^ nm^−1^. Extrapolation to a hydride volume fraction of 41% (resembling the hydride volume fraction of the 40 nm film) would result in a length of the border region of about 3.2 ∙ 10^−2^ nm^−1^. Thus, for similar volume fractions of the hydride phase, the relative length of the border regions is approximately 5 times higher for the 25 nm film as compared to the 40 nm film. This means that the length of the border region increases by reducing the film thickness.

**Figure 2:**
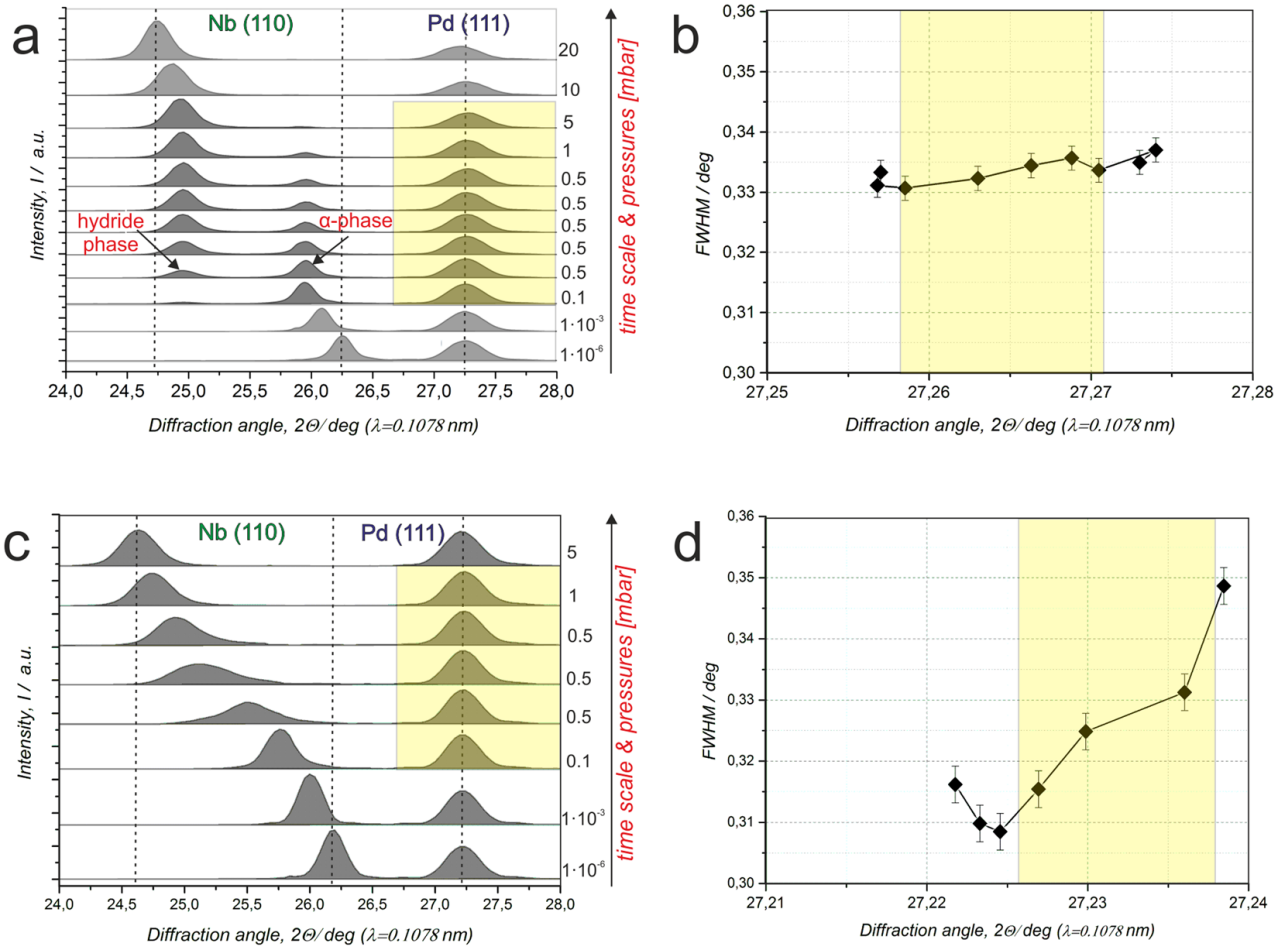
Hydrogen-induced microstructural changes measured by *in situ* XRD. (**a**,**b**) Pd20nm/Nb55nm/Al_2_0_3_ and (**c**,**d**) Pd20nm/Nb28nm/Al_2_0_3_. (**a**) Change of XRD patterns observed during hydrogen exposure at pressures of up to *p*_*H2*_ = 20 mbar (**b**) FWHM of the Pd (111) peak plotted in dependence on the peak position and the hydrogen pressure. **(c)** Change of XRD patterns observed during hydrogen exposure at pressures of up to *p*_*H2*_ = 5 mbar. Coherent phase transformation occurring within the Nb-H system is visible via the intermediate peak broadening. No separated hydride-related peak is detected. **(d)** FWHM of the Pd (111) peak plotted in dependence on the peak position. The shift of the Pd peak to higher *2θ* angles and the increase of the FWHM indicate on the stress release and an increased mosaicity of the film. (λ = 1.078 Å).

Due to the phase transformation in the Nb-film, microstructural and topographical changes will appear in the Pd layer. XRD measurements of these changes are given in Fig. 2. Figure 2(a) shows XRD patterns obtained for different stages of hydrogen exposure of a Pd20nm/Nb55nm/Al_2_O_3_ thin film system. The black arrow added on the right side of this graph provides the time axis of the experiment with arbitrary scale and the hydrogen pressure. The bottom curve shows only the Nb (110) peak at *2θ* = 26.35° and the Pd (111) peak at *2θ* = 27.25° (Fig. 2a). They correspond to the Nb-H α-phase and the Pd-H α-phase, respectively. During hydrogen exposure, the Nb-H α-phase peak shifts towards smaller angles, reflecting the lattice expansion during hydrogen absorption. Starting with the third pattern from the bottom, an additional peak appears, weak at first, at *2θ* = 24.96° corresponding to the Nb-H hydride phase. This peak’s intensity increases while the intensity of Nb-H α-phase related peak at *2θ* = 25.95° decreases, as commonly observed during the phase transformation. The phase transformation from the Nb-H α-phase to the Nb-H hydride-phase occurs in the pressure range from *p*_*H2*_ = 1 × 10^−3^ mbar to p_*H2*_ = 5 mbar. At *p*_*H2*_ = 5 mbar the phase transformation was completed as confirmed by a disappearance of the Nb-H α-phase peak. It should be noted that this hydrogen pressure is much higher than the phase transition pressure of about *p*_*H2*_ = 1 × 10^−6^ mbar found by STM measurements, even for films with similar (20 nm) Pd layer thickness. We attribute the pressure difference to the Pd surface or interface conditions that change when samples encounter environmental conditions outside the vacuum chamber. The position and the full width at half maximum (FWHM) of the Pd (111) peak at *2θ* = 27.25° change during hydrogen loading, despite hydrogen absorption occurring mainly in the Nb film (Fig. 2b). The Pd-peak shifts from 27.257° to 27.278°, while the FWHM changes from 0.333° to 0.337° (marked by yellow color). The FWHM is determined by a Gaussian fit to the Pd (111) peaks, shown in Fig. 2(a).

By further hydrogen exposure, to *p*_*H2*_ = 10 mbar and *p*_*H2*_ = 20 mbar (two upper curves in Fig. 1(a), hydrogen absorption in the Pd film (Pd-H α-phase) results in a reverse peak shift to 27.21° and an increase of the FWHM to about 0.372°. Consequently, the results of the XRD measurements suggest that the Pd layer undergoes microstructural changes resulting from the phase transformation occurring within the Nb-H film (marked by yellow color in Fig. 2(a) and (b)).

Decreasing the Nb film thickness causes different microstructural changes in the Pd film. Figure 2(c) and (d) present the results of XRD measurements on the Pd20nm/Nb28nm/Al_2_O_3_ exposed to a hydrogen gas pressure of *p*_*H2*_ ≤ 5 mbar. The phase transformation occurring in the Nb-H film causes a peak shift of the Nb (110) peak from 26.18° to 24.64°. Here, during the phase transformation only an intermediate peak broadening is detected with no separated hydride-related peak. This result is related to the coherent phase transformation in the Nb-H film^37^. At the same time, the Pd (111) peak slightly shifts from 27.221° to 27.238°. The FWHM also changes, from 0.316° to 0.349°, first decreasing from 0.316° to 0.309°, followed by an increase to 0.349°. However, the relative Pd peak shift for both samples (Fig. 2) stays almost the same and corresponds to a lattice contraction of d < 0.1%. At the same time, the change of the FWHM of the Pd peak, *ΔFWHM*, is 9 times higher for the Pd20nm/Nb28nm/Al_2_O_3_ (*ΔFWHM* = 0.0325°) than that for the Pd20nm/Nb55nm/Al_2_O_3_ (*ΔFWHM* = 0.0037°). This indicates a higher defect density in the Pd film of the Pd20nm/Nb28nm/Al_2_O_3_ system.

**Figure 3:**
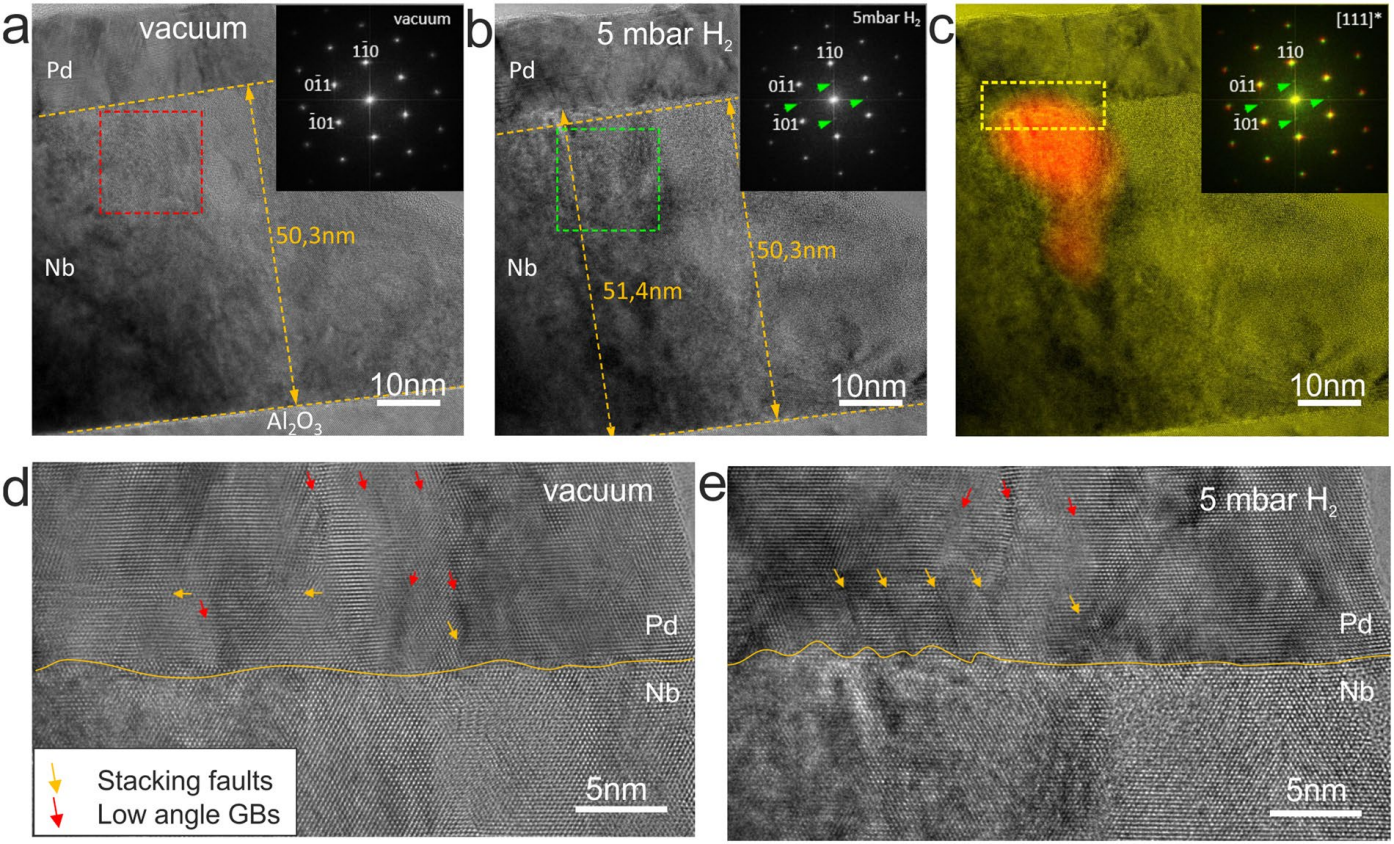
ETEM measurements on the Pd20nm/Nb50nm/Al_2_O_3_ performed *in situ* during hydrogen gas exposure. (**a**) Before hydrogen exposure (Nb layer and interfaces are marked, inset shows FFT of the rectangular region highlighted in red), (**b**) during hydrogen exposure to *p*_*H2*_ = 5 mbar (Nb layer, the original and the modified interfaces are marked, inset shows the FFT of the rectangular region highlighted in green) (**c**) precipitate of the Nb-H hydride phase localized via Fourier analysis (**d**) Pd/Nb interface before hydrogen exposure (area of interest marked in **c**) by square dashed frame, arrows show defects: stacking faults (in yellow) and low angle grain boundaries (in red). (**e**) Pd/Nb interface during hydrogen exposure to *p*_*H2*_ = 5 mbar (area of interest marked in **c**) by square dashed frames).

In order to characterize the microstructural changes in details, HRTEM measurements using cross-section specimens were performed during hydrogen gas exposure. Figure 3 shows HRTEM images of the Pd20nm/Nb50nm/Al_2_O_3_ taken before (Fig. 3a) and during hydrogen exposure at *p*_*H2*_ = 5 mbar (Fig. 3b). Fast﻿ Fourier tran﻿sform (﻿FFT) analysis of the rectangular regions highlighted in both images (see insets) clearly shows additional weak spots corresponding to an ordered hydride phase in Fig. 3(b). These spots were used to localize the hydride precipitate, as marked in red color in Fig. 3(c). The precipitate starts to grow at the Pd/Nb interface towards Nb/Al_2_O_3_ interface. The hydride phase causes the local expansion of the Nb film from 50.3 nm to 51.4 nm (Fig. 3b).

The expansion of the Nb film results in the modification of Pd/Nb interface and in the microstructural changes of the Pd film. The enlarged images of the Pd/Nb interface (selected area in Fig. 3c) before hydrogen exposure and at 5 mbar H_2_ are shown in Fig. 3(d and e), respectively. As compared to the initial position of the Pd/Nb interface marked with a yellow line in Fig. 3(d), the topographical changes of the interface of about 1–2 nm height are visible in Fig. 1(e). The interface is strongly roughened by the growth of the hydride. Moreover, the appearance of stacking faults in the Pd layer is observed close to the interface, as shown by the yellow arrows (Fig. 3(d and e)). The lateral distance between the newly formed stacking faults is similar to that between of the surface hillocks on the surface of the hydride precipitate. Additionally, the number of low angle grain boundaries decreases, as shown by red arrows in Fig. 3(d) and (e). The HRTEM images also have a typical contrast caused by the appearance of strain induced by the bending of the Pd/Nb interface Fig. 3(e).

**Figure 4:**
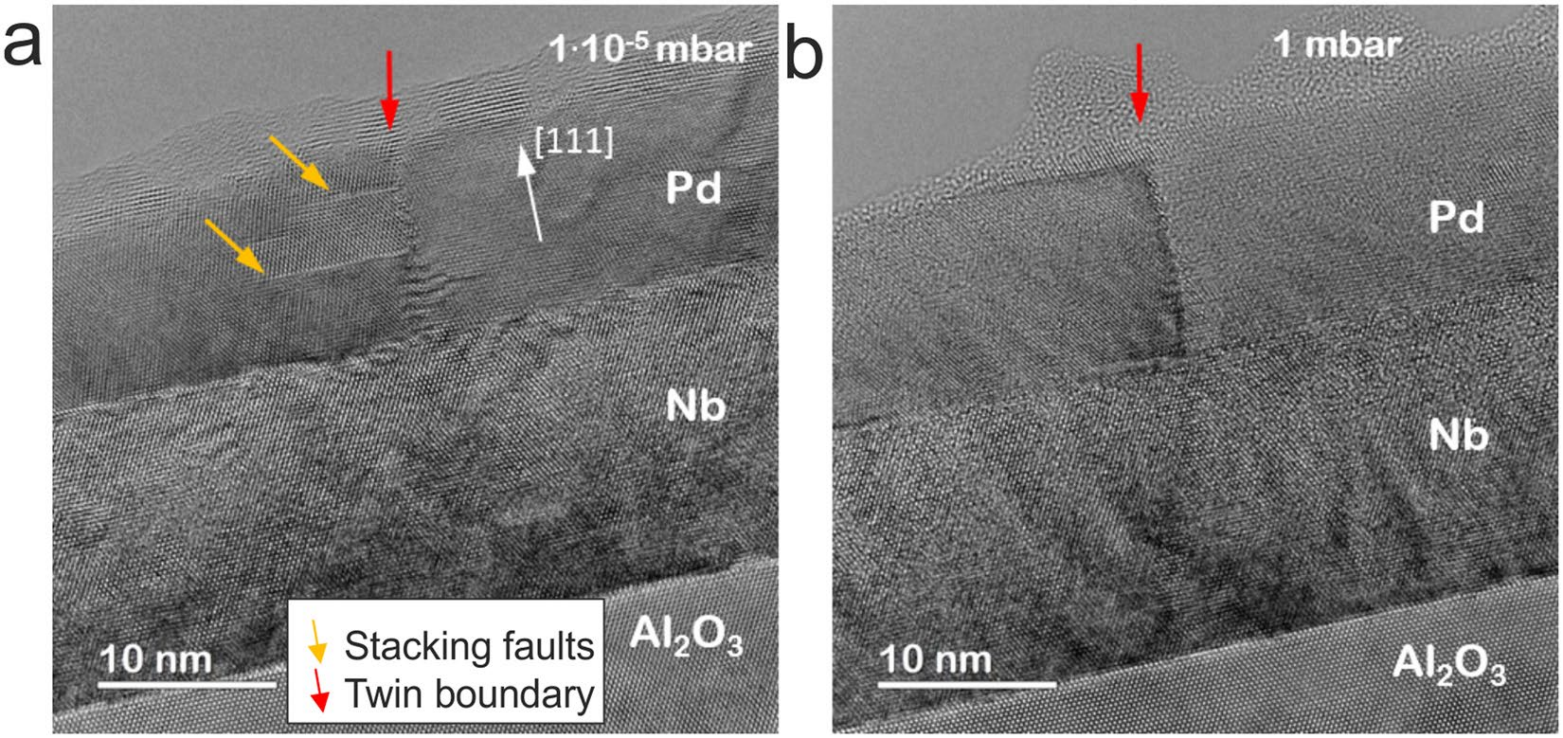
HRTEM images on the Pd12nm/Nb15nm/Al_2_O_3_. (**a**) Before the hydrogen exposure (**b**) during hydrogen exposure to *p*_*H2*_ = 1 mbar. Stacking faults presented in the area of interest before the hydrogen loading (marked with yellow arrows), disappear during the hydrogen loading with *p*_*H2*_ ≤ 1 mbar.

An additional effect that should be considered is that the Pd film also undergoes microstructural changes because of Pd interaction with H_2_. Hydrogen environment gives rise to an enhanced mobility of Pd-atoms at the surface as well as dislocation mobility and defect healing. A particular example of hydrogen-induced defect healing in Pd films is shown in Fig. 4. It shows HRTEM images of the Pd12nm/Nb15nm/Al_2_O_3_ taken before the hydrogen exposure (Fig. 4a) and during hydrogen exposure at *p*_*H2*_ = 1 mbar (Fig. 4b) in a sample area without border regions. A twin boundary between two adjacent domains (red arrow) and the stacking faults (yellow arrows) are highlighted (Fig. 4a). In the pressure range from 0.01 mbar to 0.1 mbar the stacking faults parallel to the surface of Pd film start to disappear (See Figure Sl) and they are not visible anymore at *p*_*H2*_ = 1 mbar (Fig. 4b). This result suggests that also the leading partial dislocations of the stacking faults become mobile with the presence of hydrogen. The vertical twin boundary (Fig. 4 **)** doesn’t move but its morphology is remarkably changed, as can be seen in Fig. 4(a) and (b). This may be attributed to partial dislocations being retracted into the boundary thereby removing the stacking faults. We note that the contrast change of the Nb film between Fig. 4(a) and (b) indicates a change of the stress state caused by hydrogen inside the Nb film.

**Figure 5:**
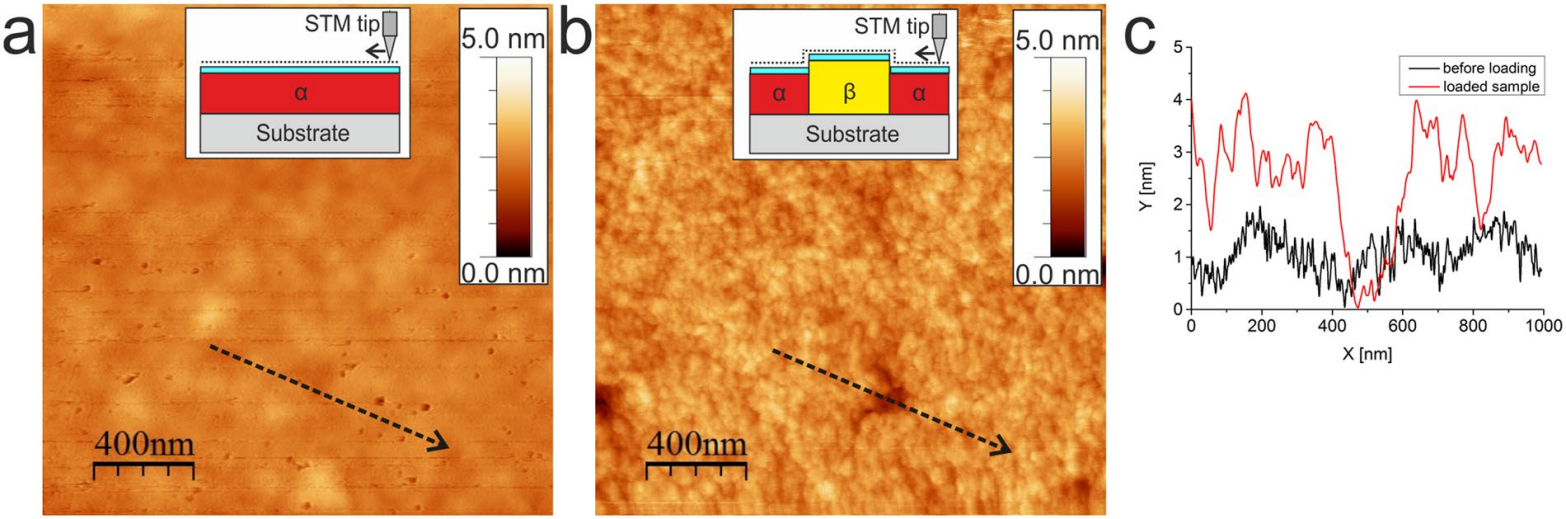
Hydrogen-induced surface topography changes of Pd2nm/Nb55nm/Al_2_O_3_ measured *in situ* by STM. (**a**) Before hydrogen exposure, (**b)** during hydrogen exposure at *p*_*H*_ = 1.6 × 10^−6^ mbar for 1220 min, (**c**) line scans taken along the black dotted lines in (**a**) and (**b**). (Frame sizes 2000 × 2000 nm^2^).

Morphological changes of Pd film may be enhanced by decreasing the thickness ratio d_Pd_/d_Nb._ A particular example with d_Pd_/d_Nb_ = 2 nm/55 nm = 0.04 is shown in Fig. 5, which summarizes STM measurements performed during hydrogen gas exposure of a Pd2nm/Nb55nm/Al_2_O_3_. Figure 5(a) shows the surface topography before hydrogen exposure, Fig. 5(b) - during hydrogen exposure at *p*_*H*_ = 1.6 × 10^−6^ mbar (*t*_*e*_ = 1220 min), and Fig. 5(c) - line scans taken along the black dotted line in (a) and (b). The STM measurements (Fig. 5b) show that the phase transition from Nb-α phase to the Nb-hydride phase was mainly completed in the loading stage, as the majority of the surface observed is expanded with respect to the initial stage. A significant surface roughening from 0.4 nm to 0.9 nm was detected upon hydrogen exposure to *p*_*H*_ = 1.6 × 10^−6^ mbar. Line scans taken along the dotted lines in Fig. 5(a) and (b) provided in Fig. 5(c) (black line: before hydrogen exposure, red line: after exposure to *p*_*H*_ = 1.6 × 10^−6^ mbar) shows the appearance of high topographic corrugations, e.g. steps and hillocks. As hydrogen-related lattice expansion in Pd is negligible at this pressure, the measured height change on the Pd-surface is mainly related to the Nb-lattice expansion.

In summary, our experiments show that the microstructural and topographic changes in the Pd-films used as a catalytic layer in Pd/Nb/Al_2_O_3_ systems are caused by the underlying Nb film and depend on its thickness. We suggest that similar effects will also show up in different multilayer systems containing hydrogen-absorbing materials. These materials can change their structure or phases depending on the environment, for example in gas sensors applications. The border regions between the different phases (present during the phase transition) appear to act as centers for bending and defect generation in the overlaying Pd-film. ‘Smart layers’ that can form hydrides may provide a means of tuning the microstructure of a coupled metal with low H affinity and may be interesting for a variety of practical applications. For example, for membrane applications the microstructural changes can be prevented by choosing an appropriate ‘smart’ layer thickness. Likewise, the microstructural features can be designed to be pronounced for the applications, such as recovery of surface activity of catalysts.

## Methods

### Material preparation

Deposition of Pd (*T*_*Sput*_ ≈ 35 °C) and Nb (*T*_*Sput*_ = 750–800 °C) films on Al_2_O_3_ (11–20) sapphire substrate is performed in an ultra-high vacuum (UHV) sputter system by cathode Argon ion-beam sputtering^30^. The thickness of the Pd layer in the Pd/Nb/Al_2_O_3_ stack is varied from 2 nm (for STM) to 20 nm (for XRD and ETEM), while the thickness of Nb layer is varied from 5 nm to 55 nm.

### Characterization

STM measurements on Pd/Nb/Al_2_O_3_ films were carried out using a UHV Micro-STM (Omicron). For hydrogen loading experiments, a Pd catalyst of 0.2 nm (islands) and a Pd film of 2 nm (closed layer) thicknesses were deposited onto Nb films. Measurements were performed directly after film preparation and without breaking the UHV conditions^30^. The STM images were obtained without changing the position of analysis (*in situ*). During experiments, the hydrogen pressure was increased in steps from 1 × 10^−9^ mbar to 5 × 10^−6^ mbar.

XRD measurements were performed on Pd/Nb/Al_2_O_3_ at the synchrotron facilities at the ESRF (Beamline BM20) and at Petra III (Beamline P08). During these measurements, the hydrogen pressure was changed stepwise from 1 × 10^−6^ mbar to 20 mbar. XRD patterns were measured in situ at different hydrogen pressures and loading times^30^.

Transmission Electron Microscopy (TEM) measurements were performed by using a FEI Titan 80–300 environmental transmission electron microscope (ETEM) operated at 300 kV, and equipped with C_s_-image corrector. During the measurements, hydrogen pressures ranging from 5 × 10^−7^ mbar to 5 mbar were applied. Most of time the electron beam was blanked to minimize its influence on the materials. For XRD and ETEM measurements, a 20 nm-thick protective Pd layer was deposited to account for sample transport in air.

## Electronic supplementary material

Defect generation in Pd layers by ‘smart’ films with high H-affinity
