# Supplementary material for: Defect generation in Pd layers by ‘smart’ films with high H-affinity
Source: Sci Rep. 2017 Aug 25;7:9564. doi: 10.1038/s41598-017-09900-z (PMC5573398; doi:10.1038/s41598-017-09900-z)
Supplement: Supplementary file 1 — Defect generation in Pd layers by ‘smart’ films with high H-affinity [file 41598_2017_9900_MOESM1_ESM.pdf]

Supplementary Information

Defect generation in Pd layers by ‘smart’ films with high H-affinity

Vladimir Burlaka, Vladimir Roddatis, Marian Bongers and Astrid Pundt

Universität Göttingen, Institut für Materialphysik, Friedrich-Hund-Platz 1, 37077 Göttingen

Sample preparation

Deposition of Pd ($T_{Sput} \approx 35\ ^{o}C$) and Nb ($T_{Sput} = 750 - 800\ ^{o}C$) films on Al_2O_3 (11-20) sapphire substrate was performed in an ultra-high vacuum (UHV) sputter system by cathode argon ion-beam sputtering ($p_{sput} = 2 \times 10^{-4}\ mbar$). For hydrogen loading experiments in the UHV-STM loading chamber, a Pd catalyst of 0.5 monolayer (islands) and 2 nm (closed layer) thickness were deposited onto Nb films.[1] For XRD and ETEM measurements, the thickness of the Pd layer was increased to about 15-20 nm in order to prevent the oxidation of the Nb film. The thickness of the Nb layer was varied in a range from 5 nm to 100 nm. XRD and pole figure measurements showed that Pd top layers exhibit texture in <111> out-of-plane orientation, while Nb films grow epitaxially on Al_2O_3 (11-20) sapphire substrate (Nb (110)//Al_2O_3 (11-20), Nb <-11-2>//Al_2O_3 <-1100>, Nb<-111>//Al_2O_3 <0001>).

STM measurements during hydrogen gas loading (in situ STM)

In situ STM measurements were carried out using an OMICRON UHV Micro-STM system (background pressure $\leq 2\times10^{-10}\ mbar$). The STM images of the same area of interest were collected continuously at different hydrogen (5.6 gas purity) pressures ($1\times10^{-9}\ mbar$ - $1\times10^{-5}\ mbar$) and exposure times (60 min – 3000 min) for subsequent data evaluation. The hydrogen pressure was adjusted via a control unit that precisely regulates the gas leak rate upon continuous pumping of the vacuum chamber. By using this loading procedure, it is possible to keep the pressure constant during the long-term STM measurements.[2]

XRD measurements during hydrogen gas loading (*in situ* XRD)

In situ XRD measurements were performed at synchrotron facilities in Grenoble (ESRF) at beam line BM20 and in Hamburg (Petra III, DESY) at beam line PO8. The portative vacuum chambers designed for in situ hydrogen gas loading experiments were used. Both chambers are equipped with automatic valves and pressure gauges which allows for a precise control of the pressure (background pressure $\leq 1\times10^{-6}\ mbar$). [1,2] The measurements were performed in the conventional θ-2θ geometry. The loading was carried out in continuous hydrogen flow, p_{H2} was

increased stepwise from $p_{H2} = 1 \times 10^{-6}$ *mbar* to $p_{H2} = 1$ *mbar*. The loading time was varied from *60 min* to *300 min* depending on the loading pressure and the film thickness. Usually, first changes in the XRD patters were observed at $p_{H2} \geq 1 \times 10^{-3}$ *mbar*.

For all the samples studied by XRD, the hydrogen gas-pressures were higher as compared to the pressure values that were required in the UHV-STM loading chamber to initiate the precipitation of the hydride phase in Nb-H thin films. We attribute this observation to a change of the sample surface or interface conditions occurring upon sample exposure to air, during the transfer to the XRD system. Comparing studies on similar Nb films covered with a 20 nm Pd film that were not exposed to air did not require higher pressures for Nb-hydride phase formation. [3]

Peak broadening upon coherent phase transformations

XRD measurements on Pd20nm/Nb28nm/Al_2O_3 did not show a separated peak related solely to the Nb hydride (**Figure 2C**). Upon hydrogen loading the Nb (110) peak shifts from *26.22* ^{o} to *24.64*^{o}. Besides, an intermediate peak broadening can be observed in the XRD pattern. The related experimental results are summarized in **Figure 1S** and **Table 1S**. The experimental curve shows that the FWHM value reaches its maximum of *0.563* ^{o} at a peak position of $2\theta = 25.155^o$ and then starts to decrease. Similar results were obtained and discussed by Burlaka et al. in detail, and attributed to the coherent phase transformation[4]. The absence of the conventionally separated α-phase and hydride phase peaks as well as the intermediate peak broadening results from several contributions: 1) coherent strains between the two adjacent phases in the two-phase region, 2) a high density of hydrides (**Figure 1f**) and 3) a low local hydrogen concentration change within the sample (width of miscibility gap gets small).

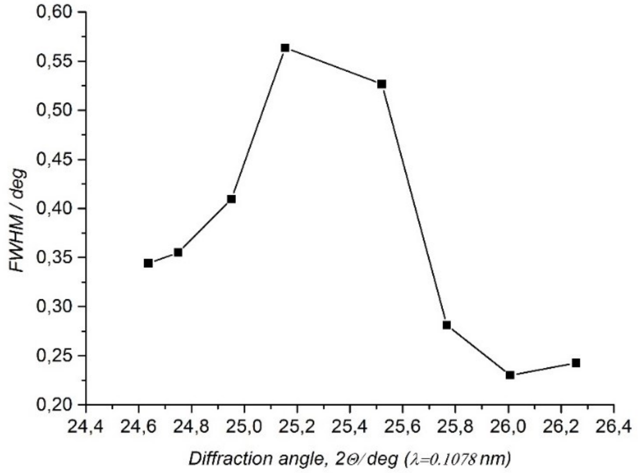

Figure 1S. FWHM of the Nb(110) peak plotted vs the peak position. The FWHM changes upon hydrogen loading of Pd20nm/Nb28nm/Al_20_3, in the two-phase region.

Table 1S. FWHM of Nb (110) peak measured at different loading stages.

Pressure, [mbar]	*Diffraction angle, 2θ / deg*	*FWHM / deg*
$1\cdot10^{-6}$	26,256	0,243
$1\cdot10^{-3}$	26,007	0,230
0.1	25,767	0,281
0.5	25,521	0,527
0.5	25,155	0,563
0.5	24,951	0,409
1	24,750	0,356
5	24,637	0,344

ETEM measurements

A FEI Nova NanoLab 600 focused ion beam (FIB) instrument was used to prepare TEM lamellas by the lift-out technique. Hydrogen-induced microstructural changes in Pd/Nb/Al_2O_3 systems were monitored using a FEI Titan 80-300 environmental transmission electron microscope (ETEM) operated at 300 keV, and equipped with energy dispersive X-Ray (EDX) and electron energy loss spectrometer (EELS). The hydrogen pressure was increased stepwise from 5×10^{-7} *mbar* to *5 mbar*. During the pressure increase and hydrogen loading (about 1 h in total) the electron beam was blanked to minimize its influence on the structure of studied films.

Defect healing in Pd film upon hydrogen exposure

The experimental HRTEM images of the Pd12nm/Nb15nm/Al_2O_3 sample at various hydrogen pressures up to 1 mbar are shown in **Figure 2S**. The hydride formation in the Nb film was not detected in the area of interest. Therefore, no interface changes occur at Pd/Nb interface. Instead, hydrogen induced defect healing is found in the Pd film. The stacking faults marked by the yellow arrows in **Figure 2S (a-e)** disappear in **Figure 2S (f).** The twin boundary between two neighboring Pd domains marked by a red arrow stays present in the area of interest. Thus, hydrogen treatment at pressures up to *1 mbar* results in defect healing occurring within the Pd layer deposited on the Nb film (high hydrogen affinity).

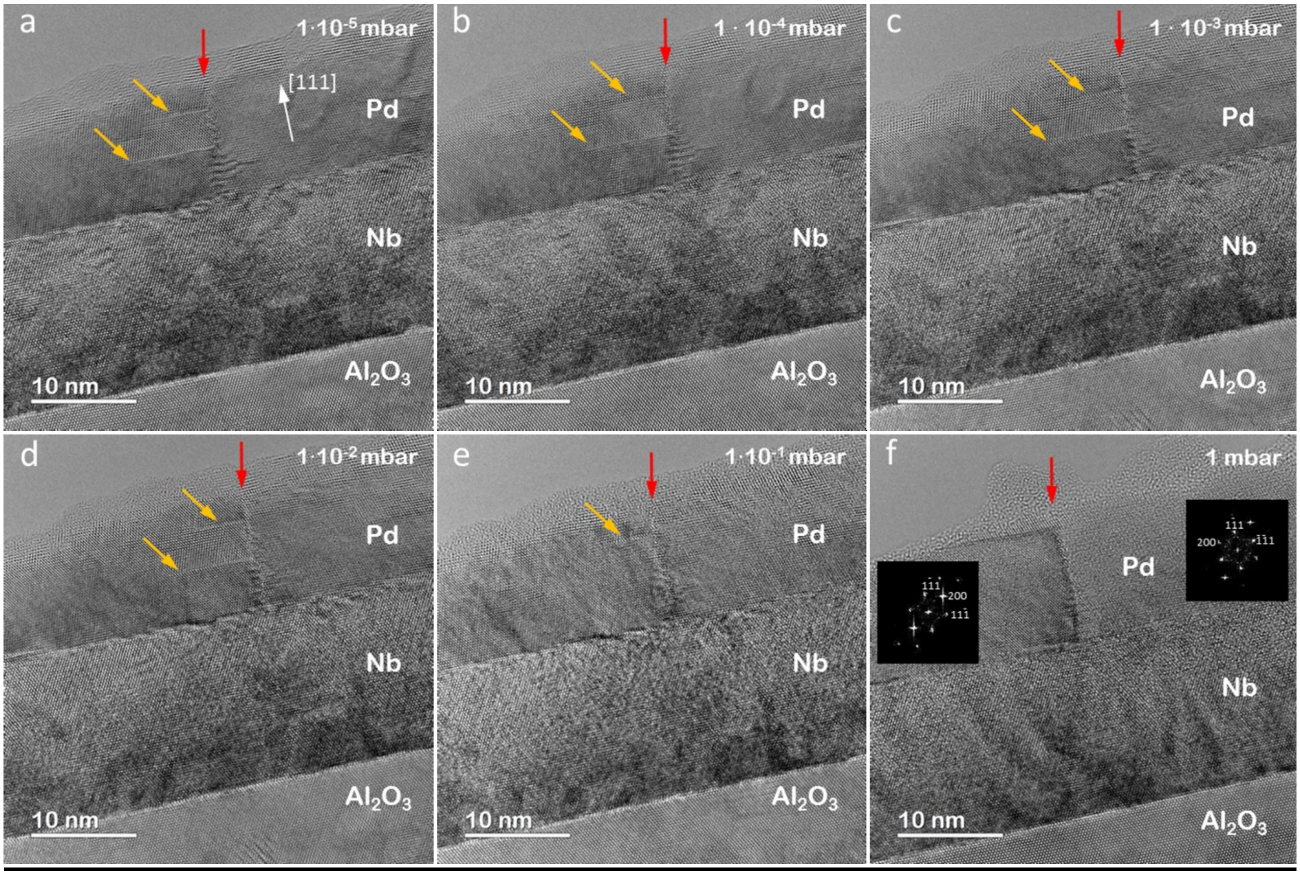

Figure 2S. HRTEM images on the Pd12nm/Nb15nm/Al_2O_3 (a) before the hydrogen exposure **(b)** during hydrogen exposure to p_{H2} = *1 mbar.* Stacking faults presented in the area of interest before the hydrogen loading (marked with yellow arrows), disappear during the hydrogen loading with $p_{H2} \leq$ *1 mbar.* The twin boundary (as confirmed by the FFT analysis) marked with a red arrow stays on its place.

In Figure 4 of the main text, a thin layer is observed above the Pd film. This film we attribute to PdC_x. By control experiments using a pure Pd foil we have observed that the formation of PdC_x does not influence the mobility of defects in Pd.

REFERENCES

1. Burlaka, V., Wagner, S., Hamm, M. & Pundt, A. Suppression of Phase Transformation in Nb-H Thin Films below Switchover Thickness. Nano Letters **16** 6207–6212 (2016).
2. Vladimir Burlaka, Critical thicknesses in Nb-H thin films: coherent and incoherent phase transitions, change of precipitation and growth modes and ultrahigh mechanical stress, PhD thesis, (Persistent Address: http://hdl.handle.net/11858/00-1735-0000-0028-87E9-A) (2015).
3. Niklas Teichmann, Bachelor thesis, *Kinetics of Hydrogen loading in epitaxial thin films of Niobium*, Göttingen (2014).
4. Burlaka, V., Wagner, S. & Pundt, A. In-situ STM and XRD studies on Nb-H films: Coherent and incoherent phase transitions. J. Alloys Compd. **645** 388-391 (2015).
